# Two new species of the *Hagnagora anicata* complex (Geometridae, Larentiinae) from Costa Rica

**DOI:** 10.3897/zookeys.149.2345

**Published:** 2011-11-24

**Authors:** J. Bolling Sullivan

**Affiliations:** 1 200 Craven Street, Beaufort, North Carolina 28516 USA

**Keywords:** Taxonomy, *Hagnagora*, Costa Rica, Colombia

## Abstract

Examination of the lectotype (here designated) reveals that *Hagnagora anicata* (Felder & Rogenhofer) does not occur in Costa Rica. Instead two new species are described, *Hagnagora elianne* **sp. n.** and *Hagnagora unnia* **sp. n.**, and their distribution is discussed. The previous treatment of *Hagnagora anicata* as a single widespread species ranging from Jamaica and Mexico to Bolivia needs to be critically evaluated.

## Introduction

Six species of the larentiine genus *Hagnagora* Druce are recorded from Costa Rica, namely *Hagnagora mortipax* (Butler), *Hagnagora clustimena* (Druce), *Hagnagora ephestris* (Felder & Rogenhofer), *Hagnagora buckleyi* Druce, *Hagnagora anicata* (Felder & Rogenhofer) and *Hagnagora marionae* Brehm & Sullivan. The most recent addition, *Hagnagora marionae*, is found at high altitudes (2500–3300 m) where it is active both diurnally and nocturnally. It can be common in night collections but is variably found sparingly during the day. The wing pattern is a band of yellow on a field of dark brown, very similar to that of *Hagnagora ephestris*, a smaller species. Both *Hagnagora anicata* and *Hagnagora mortipax* have a white wing band on the field of dark brown and again are separated by size (*Hagnagora mortipax* is smaller). Our description of *Hagnagora marionae* (Brehm and Sullivan 2005) included the ‘barcode’ sequence of the mitochondrial gene cytochrome oxidase (CO1). In order to understand the possible origin of *Hagnagora marionae* the same gene fragment was sequenced in four of the remaining five species (*Hagnagora mortipax*, *Hagnagora clustimena*, *Hagnagora ephestris*, and *Hagnagora anicata*). *Hagnagora buckleyi* in Costa Rica is known from two specimens (INBio) and no fresh material was available. These sequences showed that the closest relative of *Hagnagora marionae* was the species previously identified as *Hagnagora anicata*, a white banded species. More importantly, Costa Rican *Hagnagora anicata* separated into two haplotype groups differing in more than 5 % of their base pairs and which appeared to segregate by altitude. These findings have led to the structural and habitat characterization of the two haplotype groups and an assessment of whether or not *Hagnagora anicata*, a species described from Bogota, Colombia, was one of the two haplotype segregates.

## Materials and methods.

### Repository abbreviations

Specimens were examined from the following collections:

BMNH	Natural History Museum, London, England, UK

INBio Instituto Nacional de Biodiversidad, Santo Domingo de Heredia, Costa Rica

JBS	J. Bolling Sullivan, Beaufort, North Carolina, USA

USNM	National Museum of Natural History, Washington, District of Columbia, USA

Photographic methods used herein are described in Sullivan and Adams (2009). Procedures for dissecting and preparing genitalia follow that of Lafontaine (2004). DNA sequencing of the barcode fragment of the COI gene was carried out at the Canadian Center for DNA barcoding in Guelph, Ontario. Barcode sequences were compared by nearest neighbor analyses as implemented on the Barcode of Life Data systems website (Ratnasingham and Hebert 2007).

## Taxonomy

### Hagnagora anicata

(Felder & Rogenhofer, 1875)

http://species-id.net/wiki/Hagnagora_anicata

Fig. 1

Heterusia anicata  Felder & Rogenhofer, 1875, pl.130, fig. 13.

#### Type material.

**Figure 1.**
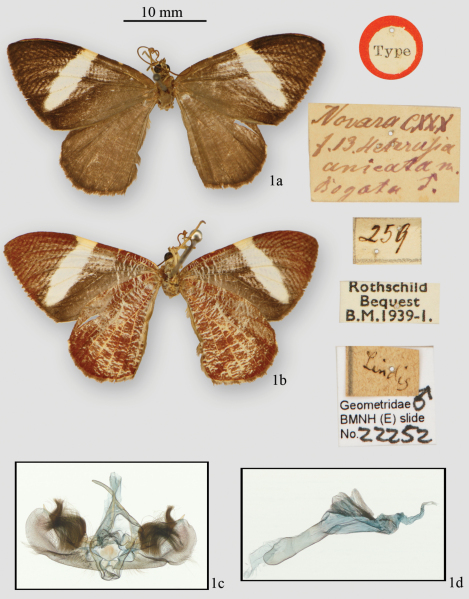
*Hagnagora anicata* (F. & R.), male lectotype **a** dorsal view **b** ventral view **c** genital capsule and **d** aedeagus (BMNH slide 22252).

Bogota, [Colombia]. A male specimen in BMNH labeled as ‘type’ is here designated as the lectotype (Fig. 1).

#### Description and diagnosis.

The maculation of *Hagnagora anicata* is shown in Fig 1a. A cream white band originates on the costa near the midpoint and runs diagonally toward a point 1/3 above the tornus, rounds off and ends without touching the margin. Distal to this band is dark brown scaling to the wing apex. Proximal to the band is dark brown scaling which becomes brighter toward the wing base. At the terminus of the wing veins are small white crescents which are usually worn off in flown specimens. The hindwing is the brownish color of the forewing base and with larger marginal crescents at the vein termini. The underside (Fig. 1b) is repeated with some slight variation in most *Hagnagora* species. This maculation pattern is seen over almost the entire range (Jamaica and Mexico to Bolivia and Venezuela), with minor modification of the width of the white band. All have been referred to as *Hagnagora anicata*.

The male genitalia are similar to those of *Hagnagora marionae* (Brehm and Sullivan 2005) and several characters are important. The uncus tapers evenly ending in a slight hook. Large hair brushes on the inner face of the valva (Fig. 1b) often obscure details of the valva, as often seen in species of another larentiine genus, *Hydriomena* Hübner. However, in *Hagnagora anicata* the costal edge of the valva is sclerotized and terminates in a well-defined point extending dorsad at the distal margin of the valva. The anal edge of the valva is swollen, stippled with small dots but not well sclerotized. The median area of the valve is unsclerotized and serves as the anchor point for the large hair brush. A smaller hair brush originates subterminally just below the costa. The juxta appears as a broad, slightly sclerotized plate and the anellus is broadened but not medially joined. The saccus is unsclerotized and u-shaped. The aedeagus is similar to that of *Hagnagora marionae* and the vesica has two dorsal, granulated wing-like projections but no cornutus.

Beside the lectotype there is a female from Bogota in the collections at the BMNH but its wing length is only 18 mm, i.e. smaller than that of the male lectotype. Because all known females of *Hagnagora* species are larger than their respective males, this female is not considered to be a female of *Hagnagora anicata*. Because there are at least two other species occurring in Colombia with maculation extremely similar to *Hagnagora anicata* (Sullivan, unpubl. data), it would be best to obtain fresh male and female specimens from near Bogota for barcode sequencing to be sure of correctly associating a female as *Hagnagora anicata*.

### Hagnagora elianne

Sullivan sp. n.

urn:lsid:zoobank.org:act:A72D314A-4993-4840-B54B-756A7C79C4A1

http://species-id.net/wiki/Hagnagora_elianne

Figs 2 4 

#### Type material.

Holotype male: **Costa Rica**, Poas Volcano National Park, Alajuela Province, 2500 m, 7–8 August 2007, J. Bolling Sullivan, DNA voucher no. 07-CRBS-1029 (INBio). **Paratypes** 9 males, 30 females: **Costa Rica.** Same data as holotype (8 males, 21 females), DNA voucher nos. 07-CRBS-383, 07-CRBS-387, 07-CRBS-389, 07-CRBS-1041, 07-CRBS-393, 07-CRBS-381, 07-CRBS-382, 07-CRBS-1042, 07-CRBS-1026, 07-CRBS-1027, 07-CRBS-1028, 07-CRBS-1031, 07-CRBS-1032, 07-CRBS-1033, 07-CRBS-1034, 07-CRBS-1035, 07-CRBS-1036, 07-CRBS-1037, 07-CRBS-1038, 07-CRBS-1046, 07-CRBS-1047, 07-CRBS-1048, 07-CRBS-1049, 07-CRBS-1050, 07-CRBS-388, 07-CRBS-391, 07-CRBS-1052, 07-CRBS-1053, 07-CRBS-1054; San Jose Province, San Gerardo de Dota, 2230 m, 16–27 March 2004, J. Bolling Sullivan, J. Donald Lafontaine, (4 females), DNA voucher nos. 06-CRBS-0050, 06-CRBS-0051, 06-CRBS-0052; Cartago Province, Villa Mills, 2841 m, J. Bolling Sullivan, (5 females), DNA voucher / GenBank accession nos. 10-CRBS-546 / HM879259, 10-CRBS-547 / HM879260, 10-CRBS-549 / HM879262, 10-CRBS-551 / HM879264, 10-CRBS-552 / HM879265; Alajuela Province, La Paz Waterfall, 1480 m. J. Bolling Sullivan (1 male). Paratypes deposited in INBio, USNM, JBS.

**Figure 2.**
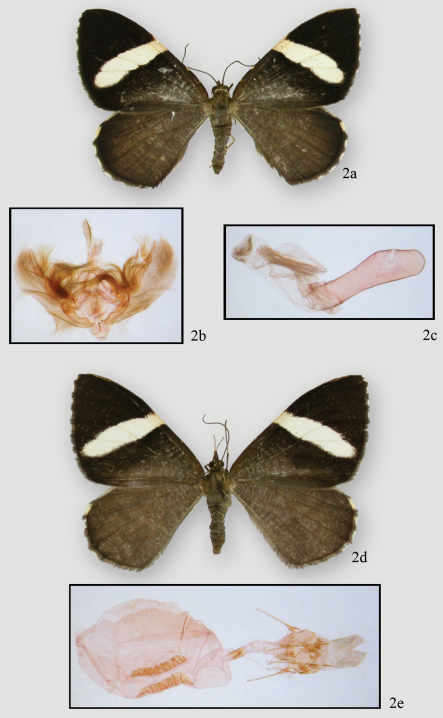
*Hagnagora elianne* Sullivan **a** adult male, holotype **b** genital capsule **c** aedeagus **d** adult female **e** female genitalia.

#### Etymology.

*Hagnagora elianne* is named for Eli-Anne Lindstrom, a scientist and friend whose biological studies of freshwater algae have contributed significantly to water quality monitoring in Norway.

#### Diagnosis.

Very similar to *Hagnagora anicata* and *Hagnagora unnia*. Males are slightly larger than those of *Hagnagora unnia* (by 2 mm on average but with overlapping ranges) but otherwise indistinguishable. They may be distinguished from *Hagnagora anicata* by the swollen distal half of the uncus (as opposed to gently tapered) and the absence of a moderately large, upcurved spine at the end of the costa. Females may be distinguished from females of *Hagnagora unnia* by their longer, more complex signa (Fig. 4). The female of *Hagnagora anicata* is undescribed. The geographical ranges of *Hagnagora elianne* and *Hagnagora anicata* are currently not known to overlap.

#### Description.

**Male**. *Head* – palps porrect, cream with brown scaling on lateral and dorsal sides of segments 2 and 3. Middle segment 2–3 x as long as other segments. Eyes round. Frons scaling slightly raised near base of tongue, brown, square, bordered with cream scales. Head brown with intermingled cream scales, particularly on edges. Collar mostly brown with scattered cream scales. Scape brown laterally and dorsally, ventral cream. Antennae simple, no visible setae, orange tan ventrally with dorsal scaling dark brown, often 70 segments. *Thorax and abdomen* – thorax brown dorsally, tegulae brown with thin cream scaling at outer edges. Abdomen brown with distal scaling on each segment forming a thin cream ring. Ventral abdomen similar, but with extensive cream scaling on basal segments. Legs mixed brown and cream scales becoming pure cream on proximal segments. Spurs on 2^nd^ and 3^rd^ tibiae small. Epiphysis reaching distal end of femur. *Wings* – forewing ( average length 19.44 mm, 18–21 mm (N=9)) with cream-white band from middle of costa traversing diagonally almost to anal angle. Distal color almost black. Proximal to diagonal band as dark as outer third of wing, then becoming brownish to base. Wing margins with thin cream crescents. Hindwing uniformly brown with terminal cream crescents at very tips. Leading edge of costa with two yellow spots evenly spaced between base and white band. Cream band on underside of forewing similar to that on upperside. Outer third of wing rust colored becoming blackish in tornal half near wing band. Basad of white band black, crossed by thin white lines. Retinaculum thumb-like, distinct, rusty. Hindwing rusty crossed by thin cream-white lines. Median area of wing (corresponding to white band on forewing) lighter, distinct. Veins distinct, orange scaled. Anal edge of forewing with white scaling. **Female.** No maculation differences from male except antennal segments thinner, retinaculum smaller, lower and more diffuse, and white scaling at ventral base of scape extends onto antennal shaft. Forewing averages 21.33 mm (20–23 mm, N=24). **Male genitalia** – terminal half of moderately long uncus distinctly swollen; tip slightly pointed but not hooked. Tegumen broad, sclerotized on lateral and proximal edges, which fuse below base of uncus, often forming a distinct X-shaped structure. Vinculum moderately broad, rather uneven on edges. Vinculum fused to segument via its proximal sclerotized edge; distal edge ends in a circular flap. Arms of anellus project finger-like to medial area but do not fuse. Juxta a large circular structure. Saccus with a broad, almost diamond-shaped structure, but often folded behind valves during slide preparation. Valva moderately broad, curves upward toward apex. Costa sclerotized but edge not well defined; ends in a small slightly upcurved spine. Valva with pronounced, swollen, medial ridge; saccular area lightly sclerotized. Medial area of valva largely unsclerotized; large hair brushes anchored near base. Medial ridge and saccular region with additional hairs. Aedeagus sclerotized to manica with out third unsclerotized, but with striations. Ductus emerges subbasally. Aedeagus with no sclerotized ridges or projections. Vesica expanded basally and forms two dorsal striated “wings.” Ventral vesica slightly striated. Vesica narrows toward terminus with dorsal surface crinkled and striated, but separate from striations of dorsal “wings.” **Female genitalia** – anal papillae gently pointed and striated toward distal tips. Posterior apophyses 1½ x length of anterior apophyses. Apophyses end proximally in a terminally-rounded paddle-like structure. Segment surfaces unsclerotized, but with tightly adhering scales. Ostial area poorly defined and unsclerotized; opening expanded laterally and tapered to moderately long and thin ductus with a proximal collar at base. Bursa an expanded sac, stippled dorsally and with two, ventral ladder-like signa. Signa look like corrugated pipes, tapering anteriorly. Length of longest sclerotized signum best character for distinguishing *Hagnagora elianne* (1.3–1.7 mm, N=8) from *Hagnagora unnia* (0.9–1.2 mm, N=4). No distinct structures on pelt. Tympanum with ansa swollen distally for half its length then narrows before expanding at proximal end to form a T-like structure with a swollen base.

### Hagnagora unnia

Sullivan sp. n.

urn:lsid:zoobank.org:act:6D9F2D75-F2BA-43D3-A056-249ED36135F6

http://species-id.net/wiki/Hagnagora_unnia

Figs 3 4 

#### Type material.

Holotype male: **Costa Rica**, Tapanti National Park, Cartago Province, 1275 m, 12–17 February 2005, J. Bolling Sullivan, DNA voucher no. 06-CRBS-0049 (INBio) **Paratypes** 13 females: **Costa Rica.** Same data as holotype (1 female), DNA voucher no. 0305-CRBS-0011; Alajuela Province, Volcan Poas, 2500 m, 7–8 August 2007, J. Bolling Sullivan, (6 females), DNA voucher nos. 07-CRBS-1040, 07-CRBS-1051, 07-CRBS-384, 07-CRBS-385, 07-CRBS-386, 07-CRBS-390; Cartago Province, Villa Mills, 2841 m, J. Bolling Sullivan, (6 females), DNA voucher / GenBank accession nos. 10-CRBS-1845 / JF856991, 10-CRBS-545 / HM879258, 10-CRBS-548 / HM879261, 10-CRBS-550 / HM879263, 10-CRBS-553 / HM879266, 10-CRBS-554 / HM879267. Paratypes deposited in INBio, USNM, JBS.

#### Etymology.

This species is named for Unni E. H. Fyhn, a postdoctoral student in my laboratory in the 1970s and who continued to work on the genetic control of fish hemoglobins until her untimely death from cancer.

#### Diagnosis.

Males are usually smaller but otherwise are indistinguishable from those of *Hagnagora elianne*. They may be distinguished from *Hagnagora anicata* by the swollen distal half of the uncus (as opposed to gently tapered) and the absence of a moderately large, upcurved spine at the end of the costa. Females may be distinguished from those of *Hagnagora elianne* by their shorter, less complex signum. The female of *Hagnagora anicata* is undescribed.

Specimens from higher altitudes are larger (see Sullivan and Miller 2007) as are most *Hagnagora elianne*. Specimens from lower altitudes are smaller as are most *Hagnagora unnia*. Although size alone cannot always be used to distinguish the species, it is often an excellent indicator, particularly where both species occur together. The expanse of the male valves (open and flattened) is often much smaller in *Hagnagora unnia*; however, this character is not always definitive. In females, the lengths of the ladder-like signa do not overlap for the two species and the sample size was larger than for males (only a single barcoded male of *Hagnagora unnia* was available). Additionally, the structure of the signum in *Hagnagora elianne* is broader and more complex.

#### Description.

**Male **. *Head* – palps porrect, cream with black scaling on lateral and dorsal sides of segments 2 and 3. Middle segment 2–3 x as long as other segments. Eyes round. Frons scaling slightly raised near base of tongue, brown, square, bordered with cream scales. Head brown with intermingled cream scales, particularly on edges. Collar mostly brown with scattered cream scales. Scape brown laterally and dorsally, ventral cream. Antenna simple, no visible setae, orange tan ventrally with dorsal scaling dark brown, often 70 segments. *Thorax and abdomen* – thorax brown dorsally; tegulae brown with thin cream scaling at outer edges. Abdomen brown with distal scaling on each segment forming a thin cream ring. Ventral abdomen similar, but with extensive cream scaling on basal segments. Legs with mixture of brown and cream scales, becoming pure cream on proximal segments. Spurs on 2^nd^ and 3^rd^ tibiae small. Epiphysis reaching distal end of tibia. *Wings* – forewing (16 mm) with cream-white band from middle of costa traversing diagonally almost to anal angle. Distal color almost black. Wing color proximal to diagonal band as dark as outer third, then becoming brown to base. Wing margins with thin cream crescents. Hindwing uniformly brown with terminal cream crescents at very tips. Leading edge of costa with two yellow spots evenly spaced between base and white band. Cream band on underside of forewing similar to that upperside. Outer third of wing rust colored becoming blackish in tornal half near wing band. Wing surface basal to white band black, crossed by thin white lines. Retinaculum thumb-like, distinct, rusty. Hindwing rusty, crossed by thin cream-white lines. Medial area of wing (corresponding to white band on forewing) lighter, distinct. Veins distinct, orange scaled. Anal edge of forewing with white scaling. **Female.** No differences from male except antennal segments thinner, retinaculum absent, and white scaling at ventral base of scape extends onto antennal shaft. Forewing length 19.33 mm (16–21mm, N=12). **Male genitalia** – terminal half of moderately long uncus distinctly swollen; tip slightly pointed but not hooked. Tegumen broad, sclerotized on lateral and proximal edges that fuse below base of uncus, often forming a distinct X-shaped structure. Vinculum moderately broad, rather uneven along its edges. Vinculum fused to tegument via its proximal sclerotized edge; distal edge ends in a circular flap. Anellar arms project finger-like to medial area but do not fuse. Juxta a large circular structure. Saccus with broad, almost diamond-shaped structure, but it often is folded behind valves during slide preparation. Valva moderately broad and curves upward toward apex. Costa sclerotized but edge is not well defined; ends in a small slightly upcurved spine. Valva with pronounced, swollen, medial ridge; sacular area lightly sclerotized. Medial area of valva largely unsclerotized, but with large hair brush anchored at base. Additional hairs are on medial ridge and saccular region. Aedeagus sclerotized to manica with out third unsclerotized, but with striations. Ductus emerges subbasally. Aedeagus without sclerotized ridges or projections. Vesica expanded basally into two dorsal striated “wings.” Ventral vesica slightly striated. Vesica narrowed toward terminus with dorsal surface crinkled and striated, but separated from striations of dorsal “wings.” **Female genitalia –** anal papillae slightly pointed and striated toward distal tips. Posterior apophyses 1½ × length of anterior apophyses; apophyses end proximally in a terminally rounded, paddle-like structure. Segment surfaces unsclerotized, but with tightly adhering scales. Ostial area poorly defined and unsclerotized. Ostial opening expanded laterally, tapered to ductus, which is moderately long and thin with a proximal collar at base. Bursa an expanded sac, stippled dorsally and with two, ventral ladder-like signa. Signa look like corrugated pipes, tapering anteriorly. Length of longest sclerotized signum is best character for distinguishing *Hagnagora elianne* from *Hagnagora unnia*. No distinct structures on pelt. Tympanum with ansa swollen distally for half its length then narrowed before expanding at proximal end to form a T-like structure with a swollen base.

**Figure 3.**
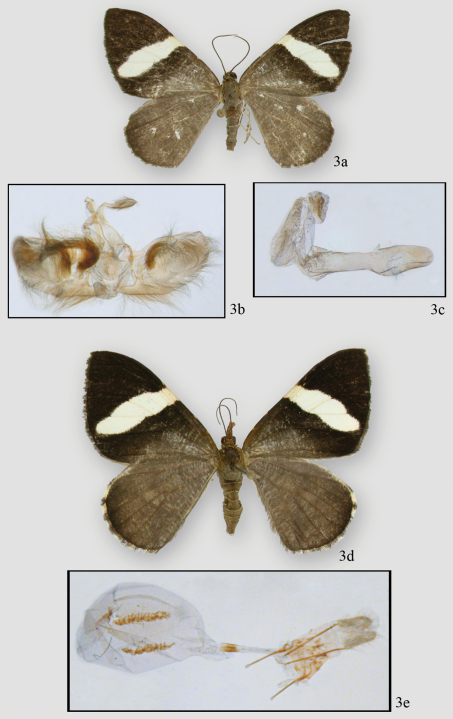
*Hagnagora unnia* Sullivan **a** adult male holotype before dissection **b** genital capsule **c** aedeagus **d** adult female **e** female genitalia.

**Figure 4.**
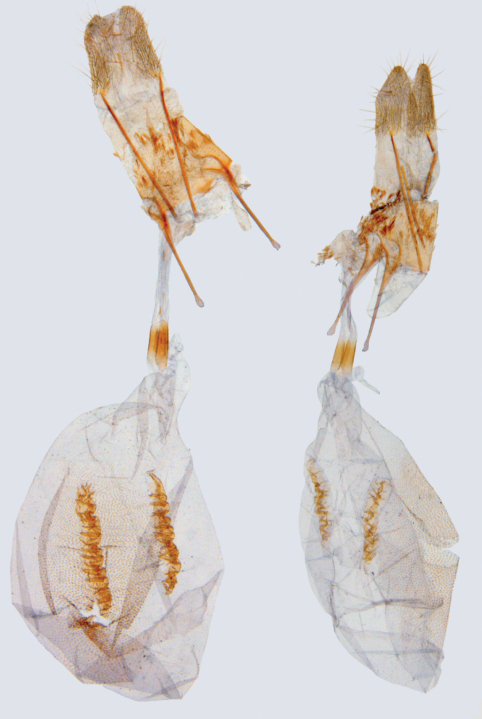
Comparison of female genitalia of *Hagnagora elianne* (left) and *Hagnagora unnia* (right) illustrating differences in length and complexity of the signa.

#### Discussion.

The *Hagnagora anicata* complex in Costa Rica illustrates a frequently occurring example in barcode work with neotropical Lepidoptera. Species with large geographical distributions are frequently a complex of species, often seemingly identical in maculation. When genitalic examination is applied to distinct barcode clusters, they usually resolve these genetic clusters, reinforcing the concept of distinct species. Additionally, other characteristics such as elevational or geographical distributions, food plant usage, and behavior, often also support the distinctness of these clusters. In the case of the *Hagnagora anicata* complex in Costa Rica, initial barcoding revealed one species above 3000 m and another below 2200 m, the species differing in more than 5% of their sequences and with little intraspecific sequence variability. When additional sampling was done at intermediate altitudes the results still supported two species. Although additional sampling needs to be done, *Hagnagora elianne* continues to occur at higher altitudes and *Hagnagora unnia* occurs at lower altitudes with the two species overlapping between 1400 and 3000 m. Based on genital characters, both species are distinct from each other and from *Hagnagora anicata*, which may not extend north of Colombia. Material from western Colombia (Valle province) differs in genital characters from *Hagnagora anicata* (Sullivan, unpubl. data) and barcoded samples from Ecuador (Brehm, unpubl. data) reveal at least two species there, both genetically distinct from the Costa Rican species. Barcoding of hundreds of species of the montane geometrid fauna from Ecuador and Costa Rica demonstrates that very few species are actually common to both regions (Brehm and Sullivan, unpubl. data).

We know little about the biology of either new species, although, based on observations at 3300 m, presumed *Hagnagora elianne* do fly diurnally, which may be true of all *Hagnagora* species (Brehm and Sullivan 2005). Known food plants used by *Hagnagora* are in the Boraginaceae (e.g., *Cordia* L.) and the Clethraceae (e.g., *Clethra* L.) (Brehm 2002) and the *Hagnagora anicata* complex likely uses the same groups of plants. Recently, King and Parra (2011) reported captive larvae of *Hagnagora vittata* (Philippi) that were reared on *Fuschia magellanica* Lamarck (Onagraceae).

The *Hagnagora anicata* complex extends from Mexico to Bolivia and east into western Venezuela paralleling the Andes. A population occurs on Jamaica. While traditionally considered a single species, barcode and genitalic analyses indicate that five or more species are probably involved in the complex. It is not unreasonable to assume that because such complexes are common in the neotropics, the estimated number of geometrid species described from there will likely continue to increase, perhaps dramatically.

## Supplementary Material

XML Treatment for Hagnagora anicata

XML Treatment for Hagnagora elianne

XML Treatment for Hagnagora unnia
